# Public Search Behavior and Tuberculosis Cases in Indonesia 2019-2023: An Infodemiology Method Using Google Trends 

**DOI:** 10.12688/f1000research.179772.1

**Published:** 2026-04-28

**Authors:** Sri Ratna Rahayu, Aufiena Nur Ayu Merzistya, Salsabila Kinaya Pranindita, Amelia Saharani, Velia Nur Ardiyani, Erna Zuliana Muanifah, Widya Hary Cahyati, Chatila Maharani, Deby Aulia Fandani, Erli Widiastuti, Aruna Daniswari, Noor Azliyana Azizan

**Affiliations:** 1 Public Health, Universitas Negeri Semarang, Semarang, Central Java, 50237, Indonesia; 2 Medicine, Universitas Negeri Semarang, Semarang, Central Java, 50237, Indonesia; 3 Centre of Physiotherapy, Universiti Teknologi MARA, Selangor, 42300, Malaysia

**Keywords:** Tuberculosis, Google Trends, Infodemiology, Search Behavior, Public Health

## Abstract

**Background:**

Tuberculosis (TB) is a major health challenge in Indonesia, which ranks second globally in 2024. As the 2030 elimination target approaches, gaps in early detection and public education persist. The public’s tendency to seek health information online before consulting professionals presents an opportunity to leverage infodemiology for public health surveillance. Therefore, this study aimed to assess the relationship between multi-term Google search trends and annual TB report data in Indonesia to disseminate the potential use of digital search data as a complementary indicator for epidemiological surveillance.

**Methods:**

A cross-sectional design was adopted to analyze the relationship between search volumes for 53 TB-related terms on Google Trends and official Indonesia Health Profile data from 2019 to 2023 across 34 provinces. Case data were normalized (0–100) to reflect the Relative Search Volume (RSV). Statistical analysis was performed using the Spearman correlation test to assess the relationship between digital searches and actual cases.

**Results:**

A consistently strong and positive correlation between TB search terms and case numbers across 34 Indonesian provinces (p < 0.001). Key correlations included “Characteristics of Pulmonary TBC ( *Ciri TBC paru*)” (r = 0.722) and “Pulmonary TBC Medicine ( *Obat TBC paru*)” (r = 0.739) in 2019, “Characteristics of Pulmonary TB ( *Ciri TB Paru*)” and “TB Prevention ( *Pencegahan TB*)” (r = 0.704) in 2020, “Characteristics of Pulmonary TB ( *Ciri TBC Paru*)” (r = 0.731) and “Childhood Pulmonary TB ( *TB Paru anak*)” (r = 0.707) in 2021, “Pulmonary TB Drugs ( *Obat TBC Paru*)” (r = 0.782) and “Characteristics of Tuberculosis ( *Ciri Tuberkulosis*)” (r = 0.709) in 2022, as well as “Characteristics of Tuberculosis ( *Ciri Tuberkulosis*)” (r = 0.731) in 2023.

**Conclusion:**

Google Trends data correlated strongly with official TB epidemiological data in Indonesia. These results suggest digital search trends can serve as complementary indicators to conventional surveillance and early warning systems.

## Introduction

TB is a chronic infection threat in the world with an estimated 10.7 million sufferers in 2024. The burden is concentrated in Southeast Asia (34%) and the Western Pacific (27%) regions. ^1, 2^ Globally, the death toll is predicted to reach 1.23 million people with a case fatality rate of 11.5%. However, control remains at 12%, a value far from the target of 50% by 2025. Indonesia consistently ranks second in terms of TB burden in the world, contributing up to 10% of the total global cases with an incidence rate of up to 382 per 100,000 people. Therefore, a wide gap remains between current conditions and the target set by Indonesian Presidential Regulation Number 67 of 2021, which aims to reduce incidence to 65 per 100,000 individuals by 2030. ^3^ Based on estimation, 1.09 million TB cases and 125 thousand deaths were recorded in the country per year. ^1^ Until August 2025, only 508,994 case notifications, or 47% of the national target, was recorded. ^4^ This gap shows that sufferers and the public tend to express their health awareness regarding TB through searching for information in digital spaces before or without accessing formal health facilities. ^5^

To determine whether search trends in digital or online spaces are in line with reality, official Indonesian government data is needed. In this case, the Indonesian Health Profile serves as a reference to present verified and validated case numbers at various levels, from regional to national. In contrast to daily data, which tends to fluctuate, the Health Profile data are compiled comprehensively to describe TB cases across the 34 provinces each year. ^6– 10^

Global health information search behavior shows that the internet, specifically through Google, has become the primary source for discovery about symptoms, diagnosis, and treatment options before consulting with a health professional. ^11, 12^ Globally, the number of internet users in 2025 will reach around 6.04 billion people, or equivalent to ±73.2% of the total population based on the Digital 2026 Global Overview Report by Data Reportal 2025. ^13^ A national survey released by the Indonesian Internet Service Providers Association (APJII) in 2025 showed that internet penetration had reached 80.66%, with the number of users being approximately 229.43 million people. ^14^ A study in Indonesia shows that the internet is used as a source of health information in the decision-making process. ^15^ Given the dominance of search engines such as Google, this platform has become a primary means of sourcing health information.

Health information activity on Google produces a digital footprint in the form of Relative Search Volume (RSV), which describes fluctuations in public interest, concerns, and pre-consultation behavior in the community. ^11, 16^ In infodemiology, this search frequency reflects a proxy for public awareness often synchronized with the number of cases in the field. ^17, 18^ The use of Google Trends to monitor infectious diseases such as TB is relevant because it can capture early signals from the population. ^19^ This digital data integration is not intended to replace conventional systems, but as a complementary tool to provide early warning of changing disease trends. ^20^

Several studies have specifically described the use of Google Trends in health studies. In a report, Google Trends data was used to analyze search volumes for infectious disease symptoms before comparing with official surveillance data to assess correlations and potential predictions of case trends. ^19^ Another study used the RSV from Google Trends related to TB in Indonesia and tested it against national notification data through correlation and time series analysis. ^5^ Google Trends data were applied in a time-lag analysis to assess whether increases in symptom-related searches preceded surges in reported cases, thereby evaluating its potential as an epidemiological early warning system. ^21^

Previous studies showed a strong correlation with the term TB (r = 0.97–1.00) and national reporting data. However, a small number of medical terms were used without comprehensively exploring the diversity of TB terminology. ^5, 22^ The present study aimed to explore 53 terms using formal, non-medical language classified into symptoms, treatment, prevention, and vulnerable groups to map the stages of search terms on Google. Correlation of multi-term data with the number of TB cases reported in the official annual Indonesian Health Profile over the past five years, hence provided a basis for evaluating the potential frequency of digital searches as a complementary indicator for more responsive epidemiological surveillance. Through an infodemiology method, the results of this study are expected to identify the most trending information in real-life cases, thereby providing an empirical basis for the development of an early warning system and targeted health communication in Indonesia.

## Methods

### Study type and design

The method used was a quantitative approach with an observational cross-sectional design. This study was adapted from previous reports related to Google Trends correlation analysis. ^23– 29^ In the process, the unit of analysis was not individuals, but provinces. The cross-sectional design was chosen to examine relationships at a single point in time, namely, each year from 2019 to 2023. This study analyzed Indonesians’ interest in Google search for information related to TB. The analysis focused on the relationship between the number of aggregated cases by province and the level of TB-related information searches on Google Trends by province during the same period. The method was chosen with the aim of determining the direction and strength of the relationship between variables.

### Data source

Data were sourced from the official annual Indonesian Health Profile reports published by the Ministry of Health for 2019, 2020, 2021, 2022, and 2023, respectively ( https://kemkes.go.id/id/category-download/profil-kesehatan ), with specific reference to the appendix, which provides the number of TB cases of all types by age group, gender, and province. The information extracted was the total number of TB cases across all genders and age groups in all 34 provinces in Indonesia. ^6– 10^

This official Ministry of Health report served as a standard value for validating web search information from Google Trends ( https://trends.google.com/trends/). The data obtained from Google Trends was the annual RSV for TB-related keywords used by the Indonesian public. All information was downloaded in comma-separated values (CSV) format on September 27, 2024. ^30^ Data from Google Trends was taken according to the annual period by province in Indonesia, starting from 2019, 2020, 2021, 2022, and 2023.

### Study variables

The dependent variable for this study was the total number of confirmed pulmonary TB cases of all types, age groups, and gender in 34 provinces across Indonesia in 2019, 2020, 2021, 2022, and 2023. The independent variable was the volume of TB-related searches on Google Trends, or the frequency of each search term. A total of 53 search terms within the Google Trends search volume, including mentions of TB, characteristics, symptoms, treatment, prevention, transmission, and TB in children and infants in 34 provinces across Indonesia in 2019, 2020, 2021, 2022, and 2023, were examined. Based on terms frequently used by Indonesians and the availability of data on Google Trends in 2019–2023, a total of 53 terms were selected. The details of the 53 terms are presented in Table 1.

**Table 1.** List of researched Google trends search terms.

Category	Term
General terms used to refer to tuberculosis in Indonesian society	*TB, TB Paru, TBC, TBC Paru, Tuberkulosis, Tuberkulosis Paru, Flek paru, Flek paru-paru * TB, Pulmonary TB, TBC, Pulmonary TBC, Tuberculosis, Pulmonary Tuberculosis, Lung spot, Lungs spot
Characteristic	*Ciri TB, Ciri TB Paru, Ciri TBC, Ciri TBC Paru, Ciri Tuberkulosis, Ciri Flek paru, Ciri Flek paru-paru * Characteristics of TB, Characteristics of Pulmonary TB, Characteristics of TBC, Characteristics of Pulmonary TBC, Characteristics of Tuberculosis, Characteristics of Lung Spot, Characteristics of Lungs Spot
Symptom	*Gejala TB, Gejala TB Paru, Gejala TBC, Gejala TBC Paru, Gejala Tuberkulosis, Gejala Tuberkulosis Paru, Gejala Flek paru, Gejala Flek paru-paru * TB Symptoms, Pulmonary TB Symptoms, TBC Symptoms, Pulmonary TBC Symptoms, Tuberculosis Symptoms, Pulmonary Tuberculosis Symptoms, Lung Spot Symptoms, Lungs Spot Symptoms
Drug	*Obat TB, Obat TB Paru, Obat TBC, Obat TBC Paru, Obat Tuberkulosis, Obat Flek paru, Obat Flek paru-paru * TB medicine, Pulmonary TB medicine, TBC medicine, Pulmonary TBC medicine, Tuberculosis medicine, Lung spot medicine, Lungs spot medicine
Prevention	*Pencegahan TB, Pencegahan TB Paru, Pencegahan TBC, Pencegahan TBC Paru, Pencegahan Tuberkulosis* TB Prevention, Pulmonary TB Prevention, TBC Prevention, Pulmonary TBC Prevention, Tuberculosis Prevention
Transmission	*Penularan TB, Penularan TB Paru, Penularan TBC, Penularan TBC Paru* TB Transmission, Pulmonary TB Transmission, TBC Transmission, Pulmonary TBC Transmission
Child	*TB anak, TB Paru anak, TBC anak, TBC paru anak, Tuberkulosis anak, Flek paru anak, Flek paru-paru anak* Childhood TB, Childhood Pulmonary TB, Childhood TBC, Childhood Pulmonary TBC, Childhood Tuberculosis, Childhood Lung Spot, Childhood Lungs Spot
Baby	*TB bayi, TB paru bayi, TBC bayi, TBC paru bayi, Tuberkulosis bayi, Flek paru bayi, Flek paru-paru bayi* Infant TB, Infant Pulmonary TB, Infant TBC, Infant pulmonary TBC, Infant tuberculosis, Infant lung spot, Infant lungs spot

TB = Tuberculosis, TBC = Tuberculosis, Lung spot = Lung spot is a term used by several Indonesian people to refer to tuberculosis.

### Data normalization and conversion

The annual aggregate of TB case count data from the official report of the Indonesian Ministry of Health from the Health Profile was converted to the same interval as the Google Trends (RSV) data, namely on a scale of 0 to 100. A scale of 0 signified no case, while 100 represented the highest number of cases in the five years from 2019 to 2023. The official report was calculated and normalized based on the annual RSV to correlate the annual aggregate TB case count report with SPSS. The following formula was used for the data normalization process ^25^:

$$\text{Normalization Formula}=\frac{\text{Highest scale}\;\left(\text{in this case}\;100\right)}{\text{Highest number of cases}}\times \text{Number of cases of the}\;i-\mathrm{th}\;\text{data}$$

### Keyword selection

Keywords were collected from Google Trends, frequently used by internet users in Indonesia. These words were grouped into eight categories, namely mentions of TB, characteristics, symptoms, treatment, prevention, transmission, tuberculosis in children, and infants.

### Statistical analysis

Data analysis used the Spearman correlation test to assess the relationship between two variables when the data were not normally distributed. Interpretation followed the criteria presented in Table 2. ^31^

**Table 2.** Interpretation of r values in correlation tests.

Absolute magnitude of observed correlation coefficient	Interpretation
0,00–0,10	Negligible correlation
0,10–0,39	Weak correlation
0,40–0,69	Moderate correlation
0,70–0,89	Strong correlation
0,90–1,00	Very strong correlation

The Spearman correlation test was used in this study to examine the relationship between search terms related to TB, characteristics, symptoms, medications, prevention, transmission, TB in children and infants, as well as the annual Indonesian Health Profile report. Furthermore, statistical analysis was performed using SPSS version 25. This study did not use human participants in the methodology, hence, ethical approval was not required.

## Results

Table 3 ( https://doi.org/10.6084/m9.figshare.31969371) explains that in 2019, analysis of 53 TB-related terms against the number of cases across 34 Indonesian provinces showed strong positive correlations for “Characteristics of Pulmonary TB ( *ciri TBC paru*)” (r = 0.722, p-value = <0.001), “Pulmonary TBC medicine ( *Obat TBC paru*)” (r = 0.739, p-value = <0.001), and “Infant pulmonary TBC ( *TBC bayi*)” (r = 0.702, p-value = <0.001). This suggested that the number of TB cases is directly proportional to the search volume for the terms.

In 2020, a strong and positive correlation between TB-related search terms and the number of TB cases in 34 Indonesian provinces was observed for “Characteristics of Pulmonary TB ( *Ciri TB Paru*)” (r = 0.704, p-value = <0.001) and “TB Prevention ( *Pencegahan TB*)” (r = 0.704, p-value = <0.001). In 2021, a strong and positive correlation between TB-related search and the number of cases in 34 Indonesian provinces was observed for “Characteristics of Pulmonary TBC ( *Ciri TBC Paru*)” (r = 0.731, p-value = <0.001) and “Childhood Pulmonary TB ( *TB Paru anak*)” (r = 0.707, p-value = <0.001). In 2022, a strong and positive correlation was observed for “Characteristics of Tuberculosis ( *Ciri Tuberkulosis*)” (r = 0.709, p-value = <0.001), “Pulmonary TBC medicine ( *Obat TBC Paru*)” (r = 0.782, p-value = <0.001), and “Childhood Pulmonary TBC ( *TBC paru anak*)” (r = 0.704, p-value = <0.001). In 2023, a strong and positive correlation was observed for “Characteristics of Tuberculosis ( *Ciri Tuberkulosis*)” (r = 0.731, p-value = <0.001).

## Discussion

In general, the analysis results in Table 3 ( https://doi.org/10.6084/m9.figshare.31969371) show a stable correlation between Google Trends and TB cases in Indonesia from 2019 to 2023, particularly for terms related to the characteristics, symptoms, transmission, and treatment. The terms tend to have moderate to strong and significantly positive correlations. This suggests that increasing cases in a region are accompanied by elevated public interest in searching for information on the signs, symptoms, and treatment. General terms such as “TB,” “TBC,” or “tuberculosis or *tuberkulosis*” have weak and unstable correlations from yearly. This signified that technical terms are less sensitive in describing the dynamics of the caseload.

Among all the keywords, the most consistently significant terms over the five years included (1) characteristics of TB, lung spot, pulmonary TB ( *ciri TB, ciri flek paru, ciri TB paru)*; (2) pulmonary TB, lung spot, tuberculosis symptoms ( *gejala TB paru, gejala flek paru, gejala tuberkulosis*), (3) TB transmission, pulmonary TB transmission, pulmonary TB transmission ( *penularan TB, penularan TB paru, penularan TBC paru*), (4) pulmonary TB, tuberculosis, lung spot medications ( *obat TBC paru, obat tuberkulosis, obat flek paru*), (5) child & infant terms such as pediatric TB, pulmonary TB in children, and lung spots in infants ( *TBC anak, TBC paru anak, flek paru bayi*). These terms tended to have stable (moderate-strong), significant correlations, and consistently reflected variations in cases across provinces.

In 2020–2021, due to the COVID-19 pandemic, respiratory-related searches generally increased, but the public’s focus was on the virus, leading to a decrease in TB-related terms. However, “lung spots ( *flek paru-paru *)” and “pulmonary TB symptoms ( *gejala TB paru*)” remained significantly correlated, reflecting strong sensitivity to the burden of this disease.

Approaching the 2022–2023 period, the correlation pattern strengthened again and showed greater consistency compared to the pandemic period. In 2022, most terms related to the characteristics, symptoms, treatment, and transmission of TB continued to show significant positive correlations with moderate to strong strength across various provinces. This was evident in keywords such as “characteristics of lung spot ( *ciri flek paru*)”, “symptoms of pulmonary TB ( *gejala TB paru*)”, “transmission of pulmonary TB ( *penularan TB paru*)”, and “medicine for lung spots ( *obat flek paru*)”, which maintained statistical significance and relatively stable correlation values. Terms related to children and infants, such as “TBC in children ( *TBC anak)*”, “pulmonary TBC in children ( *TBC paru anak*)”, and “lung spots in babies ( *flek paru bayi*)”, also showed significant correlations. This suggests that TB-related searches in vulnerable age groups are increasingly sensitive to variations in caseload.

In 2023, the strengthening trend in correlations became even more evident, particularly for clinical and specific terms. Several keywords, such as “characteristics of pulmonary TB ( *ciri TB Paru*)”, characteristics of lung spots ( *ciri flek paru*), symptoms of lung spots ( *gejala flek paru*), TB transmission ( *penularan TB*), and pulmonary TB medication ( *obat TB paru*), showed significant correlations with consistent values in the moderate to strong category. This suggested that after a period of disruption to health services due to COVID-19, individuals are seeking more specific and targeted TB information. Conversely, general terms such as “TB” or “TBC” continue to show weak and inconsistent correlations, becoming less representative as digital indicators for monitoring the burden of the disease. The 2022–2023 period confirms that symptomatic, diagnostic, and therapeutic keywords have higher sensitivity in reflecting variations in cases across regions than general terms.

A correlation analysis of Google Trends for TB cases in Indonesia shows that keywords based on symptoms, clinical characteristics, transmission, and treatment have a more stable relationship than general terms. As a result, the keywords are potentially being used as digital indicators to show spatio-temporal disease dynamics. Several infodemiology-based studies have shown that TB-related search volume on search engines significantly reduces cases and can reflect epidemiological trends. A study in South Africa showed a significant positive correlation between Google Trends search volume and reported incidence rates. Eight search terms showed moderate to strong associations, including “tuberculosis” and “TB,” as well as searches related to symptoms and diagnostic tests such as the “Mantoux test.” Furthermore, a strong correlation pattern was observed for terms related to comorbidities, particularly diabetes and HIV, reflecting the established pattern of HIV-TB infection in the region and increasing public awareness of the risk of diabetes to TB. ^32^

Another study used Google Trends to correlate measles clinical cases using Pearson correlation in 30 European countries and Japan. The results showed a very strong correlation at the regional level in developed countries, as observed in Okinawa Prefecture, Japan, during the 2017–2019 period. This is much higher than the correlation at the national level, proving that digital data searches are much more accurate in capturing signals of specific outbreaks in specific locations than on a broader scale. Search behavior is more sensitive to acute outbreaks that appear suddenly with many cases in a short period. Conversely, prolonged outbreaks with few cases per week often fail to be captured by Google Trends because individuals tend to no longer actively search for information when the disease is considered normal or not widespread. ^33^

Search for health information is often triggered by the development of disease symptoms that then trigger anxiety. When certain symptoms are experienced, search engines might be used to conduct initial identification before seeking professional medical help. ^34^ This behavior leads to a significant increase in search volume for symptom-related keywords, which often precedes official reports from health surveillance systems. The phenomenon suggests that digital search data can serve as an early indicator of public reaction to developing health threats. ^35^

Search volume tends to increase sharply in areas with a high disease burden or during outbreaks. In Indonesia, studies on dengue fever and COVID-19 have shown a strong correlation between the number of actual cases and search trends for keywords such as “symptoms” or “transmission”. ^36^ This linear pattern shows that individuals in affected areas are actively sourcing information for self-diagnosis or preventative measures. Therefore, a spike in searches in a specific region signals to health authorities the presence of potential disease hotspots requiring immediate intervention. ^25^

Pandemics, such as COVID-19, can create an overshadow effect that disrupts the seasonal patterns of other infectious diseases. During the peak of a pandemic, public attention and medical resources are highly focused on the novel virus. This leads to a decrease or change in search behavior toward other diseases, such as RSV or influenza. After pandemic mitigation measures are relaxed, search patterns and outbreaks of other diseases often reappear at irregular times and with greater intensity. This emphasizes the importance of using digital monitoring tools such as Google Trends to stay aware of multiple health risks amid the dominance of a single large outbreak. ^35^

The present study has several limitations, including its reliance on internet access, which is not evenly distributed across Indonesia. This implies that Google Trends data may be more representative of the behavior of residents in urban areas than in rural areas. Furthermore, the intent behind keyword searches cannot be fully ascertained, whether being performed by TB patients for treatment or by healthy individuals simply sourcing general information. The study also did not include user demographic factors such as age and education level, which may influence how search terms are formulated in search engines.

## Conclusion

In conclusion, digital search data through Google Trends had a significant positive correlation with the burden of TB cases in Indonesia, offering significant potential as a complementary epidemiological surveillance (infodemiology) instrument. Key results showed that the public was more prone to use non-medical or popular terms such as “lung spots” and specific keywords related to “characteristics,” “symptoms,” and “treatment” than formal technical terms. The consistent, strong correlation across children and infants also confirmed high demand for digital information among vulnerable groups. These search patterns reflected real-world public health behavior, which, when integrated with official reporting systems, could strengthen early warning systems and support more targeted health communication strategies aimed at achieving the 2030 TB elimination target.

### Implications and recommendations

The results of this study provide an empirical basis for policymakers to integrate Google Trends data as an early warning system to complement conventional surveillance systems. Public health communication strategies should be optimized by using popular terms in the community, such as “lung spot ( *flek paru*)”. As a result, educational messages are more targeted and effective in encouraging early detection to achieve the national TB elimination target by 2030.

## Data Availability

Figshare: Public Search Behavior and Tuberculosis Cases in Indonesia 2019–2023: An Infodemiology Method Using Google Trends, https://doi.org/10.6084/m9.figshare.31969344 ^37^ The project contains the following underlying data:
• Data.xlsx. (Tuberculosis Case Data and Google Trends Search Data) Data are available under the terms of the Creative Commons Attribution 4.0 International license (CC-BY 4.0). Figshare: Public Search Behavior and Tuberculosis Cases in Indonesia 2019–2023: An Infodemiology Method Using Google Trends, https://doi.org/10.6084/m9.figshare.31969371 ^37^ This project sontaints the following extended data:
•
Table 3. The relationship between the 53 TB search terms on Google Trends and number of TB cases in Indonesia 2019–2023 Data are available under the terms of the Creative Commons Attribution 4.0 International license (CC-BY 4.0).
