# Public Search Behavior and Tuberculosis Cases in Indonesia 2019-2023: An Infodemiology Study Using Google Trends 

**DOI:** 10.12688/f1000research.179772.2

**Published:** 2026-09-12

**Authors:** Sri Ratna Rahayu, Aufiena Nur Ayu Merzistya, Salsabila Kinaya Pranindita, Amelia Saharani, Velia Nur Ardiyani, Erna Zuliana Muanifah, Widya Hary Cahyati, Chatila Maharani, Deby Aulia Fandani, Erli Widiastuti, Aruna Daniswari, Noor Azliyana Azizan

**Affiliations:** 1 Public Health, Universitas Negeri Semarang, Semarang, Central Java, 50237, Indonesia; 2 Medicine, Universitas Negeri Semarang, Semarang, Central Java, 50237, Indonesia; 3 Centre of Physiotherapy, Universiti Teknologi MARA, Selangor, 42300, Malaysia

**Keywords:** Tuberculosis, Google Trends, Infodemiology, Search Behavior, Public Health

## Abstract

**Background:**

Tuberculosis (TB) remains a major health challenge in Indonesia, which ranks second globally in 2024. As the 2030 elimination target approaches, gaps in early detection and public education persist. The public’s tendency to seek health information online before consulting professionals presents an opportunity to leverage infodemiology for public health surveillance. Therefore, this study aimed to assess the relationship between multi-term Google search trends and annual TB report data in Indonesia to disseminate the potential use of digital search data as a complementary indicator for epidemiological surveillance.

**Methods:**

A repeated cross-sectional design was conducted using provincial data from all Indonesian provinces between 2019 and 2023. Official provincial TB case data were obtained from the Indonesian Health Profile, while Google Trends Relative Search Volume (RSV) data were extracted for 53 TB-related search terms. Provincial TB case counts were normalized to a 0–100 scale to match RSV values before analysis. Normality was assessed using the Shapiro–Wilk test. The associations between TB cases and RSV were examined using Spearman’s rank correlation. Multiple testing was controlled using the Benjamini–Hochberg False Discovery Rate (BH-FDR) procedure, with statistical significance defined as an FDR-adjusted *p* < 0.05.

**Results:**

After false discovery rate adjustment, 38–42 of the 53 tuberculosis-related Google Trends search terms remained significantly correlated with monthly TB cases across 2019–2023, indicating a stable association between online search behavior and disease incidence. Colloquial search terms (e.g., “Flek Paru”), clinical characteristic queries, and keywords related to symptoms, prevention, transmission, treatment, and pediatric TB consistently showed the strongest positive correlations, with correlation coefficients reaching *r* = 0.731 (all adjusted *p* < 0.05).

**Conclusion:**

Google Trends data, correlated strongly with TB case in Indonesia, can complement conventional TB surveillance by reflecting regional disease patterns and supporting timely monitoring, despite limitations related to internet access and search behavior.

## Introduction

Tuberculosis (TB) is a chronic infection threat in the world with an estimated 10.7 million sufferers in 2024. The burden is concentrated in Southeast Asia (34%) and the Western Pacific (27%) regions. ^1^ Globally, the death toll is predicted to reach 1.23 million people with a case fatality rate of 11.5%. However, only 12% reduction in TB incidence rate, a value far from the target of 50% by 2025. Indonesia consistently ranks second in terms of TB burden in the world, contributing up to 10% of the total global cases with an incidence rate of up to 382 per 100,000 people. Therefore, a wide gap remains between current conditions and the target set by Indonesian Presidential Regulation Number 67 of 2021, which aims to reduce incidence to 65 per 100,000 individuals by 2030. ^2^ Based on estimation, 1.09 million TB cases and 125 thousand deaths were recorded in the country per year. ^1^ Until August 2025, only 508,994 case notifications, or 47% of the national target, was recorded. ^3^ This gap may indicate increased public reliance on digital platforms to seek TB-related health information. ^4^

To determine whether search trends in digital or online spaces are in line with reality, official Indonesian government data is needed. In this case, the Indonesian Health Profile serves as a reference to present verified and validated case numbers at various levels, from regional to national. In contrast to daily data, which tends to fluctuate, the Health Profile data are compiled comprehensively to describe TB cases across the 34 provinces each year. ^5^

Global health information search behavior shows that the internet, specifically through Google, has become the primary source for discovery about symptoms, diagnosis, and treatment options before consulting with a health professional. ^6, 7^ Globally, the number of internet users in 2025 will reach around 6.04 billion people, or equivalent to ±73.2% of the total population. ^8^ A national survey in 2025 showed that internet penetration had reached 80.66%, with the number of users being approximately 229.43 million people. ^9^ A study in Indonesia shows that the internet is used as a source of health information in the decision-making process. ^10^ Given the dominance of search engines such as Google, this platform has become a primary means of sourcing health information.

In the Indonesian setting, Google Trends has the potential to reflect TB burden because individuals often seek health information online when experiencing symptoms suggestive of TB or when exposed to information from healthcare providers, family members, or media. With Google serving as the dominant search engine and internet penetration exceeding 80% of the population, changes in TB-related search activity may correspond to changes in public awareness and healthcare-seeking behaviour associated with disease occurrence. A recent study in Indonesia demonstrated strong correlations between Google Trends data and national TB notification data both before and after the implementation of the mandatory TB notification system, supporting the potential of Google Trends as a complementary indicator for TB surveillance. ^4^

Health information activity on Google produces a digital footprint in the form of Relative Search Volume (RSV), which describes fluctuations in public interest, concerns, and pre-consultation behavior in the community. ^6, 11^ In infodemiology, this search frequency reflects a proxy for public awareness often synchronized with the number of cases in the field. ^12, 13^ The use of Google Trends to monitor infectious diseases such as TB is relevant because it can capture early signals from the population. ^14^ This digital data integration is not intended to replace conventional systems, but as a complementary tool to provide early warning of changing disease trends. ^15^

Google Trends data was used to analyze search volumes for infectious disease symptoms before comparing with official surveillance data to assess correlations and potential predictions of case trends. ^14^ Recent studies have also highlighted the broader application of digital health technologies in strengthening TB surveillance. The implementation of electronic medical record systems has been shown to improve the accuracy, completeness, and accessibility of TB surveillance data, thereby supporting more efficient disease monitoring and patient follow-up in high-burden settings. ^16^ Similarly, a study conducted in Indonesia reported a strong correlation between Google Trends data and national TB notification data, supporting the potential of online search behaviour as a complementary indicator for monitoring TB trends. This study used the RSV from Google Trends related to TB in Indonesia and tested it against national notification data through correlation and time series analysis. ^4^ Google Trends data were applied in a time-lag analysis to assess whether increases in symptom-related searches preceded surges in reported cases, thereby evaluating its potential as an epidemiological early warning system. ^17^ However, a small number of medical terms were used without comprehensively exploring the diversity of TB terminology. ^4^

This study was to evaluate the correlation between Google Trends search volumes and reported TB cases in Indonesia from 2019 to 2023 using various Indonesian-language search terms, encompassing disease names, symptoms, clinical characteristics, treatment, prevention, transmission, and pediatric TB. Correlation of multi-term data with the number of TB cases reported in the official annual Indonesian Health Profile over the past five years, hence provided a basis for evaluating the potential frequency of digital searches as a complementary indicator for more responsive epidemiological surveillance. Unlike previous studies that typically relied on a limited number of generic keywords, this study identifies culturally relevant search terms that more accurately reflect TB trends in Indonesia and provides evidence to support the development of locally adapted digital disease surveillance strategies. Through an infodemiology method, the results of this study are expected to identify the most trending information in real-life cases, thereby providing an empirical basis for the development of an early warning system and targeted health communication in Indonesia.

## Methods

### Study type and design

This study employed a quantitative ecological study using repeated cross-sectional provincial data collected annually from 2019 to 2023. This study was adapted from previous reports related to Google Trends correlation analysis. ^18– 24^ In the process, the unit of analysis was not individuals, but provinces. The cross-sectional design was chosen to examine relationships at a single point in time, namely, each year from 2019 to 2023. This study analyzed Indonesians’ interest in Google search for information related to TB. The analysis focused on the relationship between the number of aggregated cases by province and the level of TB-related information searches on Google Trends by province during the same period. The method was chosen with the aim of determining the direction and strength of the relationship between variables.

### Data source

Data were sourced from the official annual Indonesian Health Profile reports published by the Ministry of Health for 2019–2023, respectively, with specific reference to the appendix, which provides the number of TB cases of all types by age group, gender, and province. The information extracted was the total number of TB cases across all genders and age groups in all 34 provinces in Indonesia. ^5^

This official Ministry of Health report served as a standard value for validating web search information from Google Trends ( https://trends.google.com/trends/). The data obtained from Google Trends was the annual RSV for TB-related keywords used by the Indonesian public. All information was downloaded in comma-separated values (CSV) format on September 27, 2024. ^25^ Data from Google Trends was taken according to the annual period by province in Indonesia, starting from 2019 until 2023.

Google Trends data were extracted using Web Search with the geographic filter set to Indonesia. All search terms were entered in the Indonesian language, the search category was set to All Categories, and the search type was Web Search. Data extraction was performed separately for each study year using annual time intervals (January 1 ^st^ to December 31 ^st^) for 2019–2023. Each TB-related search term was queried individually for each study year. Consequently, each search term generated five separate CSV files (one file for each year), and each CSV file contained the Interest by subregion results, consisting of RSV values for all 34 Indonesian provinces. Province-specific RSV values from each annual CSV file were then matched with the corresponding provincial TB case data for the same year obtained from the Indonesian Health Profile before Spearman correlation analysis.

### Study variables

The dependent variable for this study was the total number of confirmed pulmonary TB cases of all types, age groups, and gender in 34 provinces across Indonesia in 2019–2023. The independent variable was the volume of TB-related searches on Google Trends, or the frequency of each search term. A total of 53 search terms within the Google Trends search volume, including mentions of TB, characteristics, symptoms, treatment, prevention, transmission, and TB in children and infants in 34 provinces across Indonesia in 2019–2023, were examined. Based on terms frequently used by Indonesians and the availability of data on Google Trends in 2019–2023, a total of terms were selected. The details of the terms are presented in Table 1.

**Table 1.** List of researched Google trends search terms.

Category	Term
General terms used to refer to tuberculosis in Indonesian society	*TB, TB Paru, TBC, TBC Paru, Tuberkulosis, Tuberkulosis Paru, Flek paru, Flek paru-paru * TB, Pulmonary TB, TBC, Pulmonary TBC, Tuberculosis, Pulmonary Tuberculosis, Lung spot, Lungs spot
Characteristic	*Ciri TB, Ciri TB Paru, Ciri TBC, Ciri TBC Paru, Ciri Tuberkulosis, Ciri Flek paru, Ciri Flek paru-paru * Characteristics of TB, Characteristics of Pulmonary TB, Characteristics of TBC, Characteristics of Pulmonary TBC, Characteristics of Tuberculosis, Characteristics of Lung Spot, Characteristics of Lungs Spot
Symptom	*Gejala TB, Gejala TB Paru, Gejala TBC, Gejala TBC Paru, Gejala Tuberkulosis, Gejala Tuberkulosis Paru, Gejala Flek paru, Gejala Flek paru-paru * TB Symptoms, Pulmonary TB Symptoms, TBC Symptoms, Pulmonary TBC Symptoms, Tuberculosis Symptoms, Pulmonary Tuberculosis Symptoms, Lung Spot Symptoms, Lungs Spot Symptoms
Drug	*Obat TB, Obat TB Paru, Obat TBC, Obat TBC Paru, Obat Tuberkulosis, Obat Flek paru, Obat Flek paru-paru * TB medicine, Pulmonary TB medicine, TBC medicine, Pulmonary TBC medicine, Tuberculosis medicine, Lung spot medicine, Lungs spot medicine
Prevention	*Pencegahan TB, Pencegahan TB Paru, Pencegahan TBC, Pencegahan TBC Paru, Pencegahan Tuberkulosis* TB Prevention, Pulmonary TB Prevention, TBC Prevention, Pulmonary TBC Prevention, Tuberculosis Prevention
Transmission	*Penularan TB, Penularan TB Paru, Penularan TBC, Penularan TBC Paru* TB Transmission, Pulmonary TB Transmission, TBC Transmission, Pulmonary TBC Transmission
Child	*TB anak, TB Paru anak, TBC anak, TBC paru anak, Tuberkulosis anak, Flek paru anak, Flek paru-paru anak* Childhood TB, Childhood Pulmonary TB, Childhood TBC, Childhood Pulmonary TBC, Childhood Tuberculosis, Childhood Lung Spot, Childhood Lungs Spot
Baby	*TB bayi, TB paru bayi, TBC bayi, TBC paru bayi, Tuberkulosis bayi, Flek paru bayi, Flek paru-paru bayi* Infant TB, Infant Pulmonary TB, Infant TBC, Infant pulmonary TBC, Infant tuberculosis, Infant lung spot, Infant lungs spot

TB = Tuberculosis, TBC = Tuberculosis, Lung spot = Lung spot is a term used by several Indonesian people to refer to tuberculosis.

### Data normalization and conversion

To facilitate direct comparison between annual provincial TB case counts and Google Trends RSV, the provincial TB case counts obtained from the Indonesian Health Profile were normalized to the same 0–100 scale as the RSV data, following the approach used in previous Google Trends surveillance studies. Normalization was performed separately for each study year (2019–2023) using annual maximum normalization. For each year, the province with the highest reported TB case count was assigned a normalized score of 100, while the remaining provinces were proportionally rescaled relative to the highest provincial TB case count recorded in the same year. The normalization procedure was performed because official TB case data and Google Trends’ RSV are reported on different numerical scales. The annual provincial TB case counts obtained from the Indonesian Health Profile are recorded as absolute numbers (ranging from thousands to over one hundred thousand cases), while Google Trends reports RSV on a standard scale of 0 to 100.The following formula was used for the data normalization process ^20^:

$$\text{Normalization Formula}=\frac{\text{Highest scale}\;\left(\text{in this case}\;100\right)}{\text{Highest provincial cases count}}\times {\text{Provincial TB case count}}_{\;i}$$

Where:

- 100 = maximum normalized score
- Highest provincial TB case count = the largest provincial TB case count in the corresponding year
- Provincial TB case count _*i*_ = reported TB cases for province *i*

For example, the highest provincial TB case count was reported in West Java (123,021 cases) in 2019, which was assigned a normalized score of 100. Aceh reported 8,396 TB cases in the same year; therefore, its normalized score was calculated as:

$$\text{Normalization Formula}=\frac{100}{123.021}\times 8.396=6,82$$

Accordingly, Aceh received a normalized TB score of 7, while West Java received a score of 100. This normalization procedure was repeated independently for each study year (2019–2023), ensuring that each year’s provincial TB case counts were rescaled relative to the annual maximum before being paired with the corresponding Google Trends RSV values for Spearman correlation analysis.

### Keyword selection

Keywords were collected from Google Trends, frequently used by internet users in Indonesia. These words were grouped into eight categories, namely mentions of TB, characteristics, symptoms, treatment, prevention, transmission, TB in children, and infants.

### Statistical analysis

Data were first assessed for normality using the Shapiro–Wilk test because each annual dataset consisted of 34 provincial observations. The results indicated that both provincial TB case counts and Google Trends RSV data were not normally distributed across all study years (2019–2023) in Table 3. ^20^ Therefore, the Spearman’s rank correlation test was used to assess the association between provincial TB cases and RSV for each TB-related search term. The strength of the correlation was interpreted according to the criteria presented in Table 2. ^26^

**Table 2.** Interpretation of r values in correlation tests.

Absolute magnitude of observed correlation coefficient	Interpretation
0,00–0,10	Negligible correlation
0,10–0,39	Weak correlation
0,40–0,69	Moderate correlation
0,70–0,89	Strong correlation
0,90–1,00	Very strong correlation

Multiple testing correction was performed using the Benjamini–Hochberg False Discovery Rate (BH-FDR) procedure to control the expected false discovery rate while maintaining greater statistical power than conventional family-wise error rate correction methods because 53 correlation analyses were conducted independently for each study year. Following the Benjamini–Hochberg procedure, the raw *P*-values were ranked in ascending order, and the preliminary adjusted value for each test was calculated as:

$$P_{\mathrm{BH}\;\left(i\right)}=\frac{P\;\left(i\right)\times m}{i}$$

where *P*( *i*) is the ordered raw *P*-value, *i* is the rank of the *P*-value after sorting in ascending order, and *m* is the total number of hypothesis tests (53 search terms evaluated for each study year).

To ensure monotonicity of the adjusted *P*-values, the preliminary BH values were subsequently corrected sequentially from the largest to the smallest rank using ^27, 28^:

$$P_{\left(i\right)}^{\mathrm{adj}}=\min\;(P_{\mathrm{BH}\left(i\right),P_{\left(i+1\right)}^{\mathrm{adj}}})$$

The BH-FDR calculations were implemented in Microsoft Excel, whereas the Spearman correlation coefficients and raw *P*-values were obtained using IBM SPSS Statistics version 25. Statistical significance was defined as an FDR-adjusted *P* < 0.05. This study used only publicly available aggregated provincial surveillance data and Google Trends data and therefore did not involve human participants or require ethical approval.

## Results

The correlations between monthly Google Trends search volumes for 53 TB-related search terms and the reported number of TB cases in Indonesia from 2019 to 2023 are presented in Table 4. The most of search terms demonstrated statistically significant correlations with TB incidence throughout the study period, after controlling for multiple comparisons using the False Discovery Rate (FDR). The number of search terms that remained statistically significant, with 39 terms in 2019, 42 in 2020, 40 in 2021, 40 in 2022, and 38 in 2023. This stability suggests a consistent relationship between public online search behavior and TB case.

Search terms in colloquial language showed stronger correlations than those using formal medical terminology, such as “Flek paru-paru”. In particular “Flek paru” and “Flek paru-paru” consistently demonstrated moderate-to-strong positive correlations throughout the study period (r = 0.468–0.639; all FDR-adjusted p < 0.01). Other terms, such as “Tuberkulosis Paru” were significantly moderate-to-weak correlations with TB cases from 2019 to 2022 (r = 0.372–0.590), whereas broader terms such as “TB” and “TBC” exhibited weak or inconsistent associations.

Terms related to clinical characteristics produced some of the strongest correlations observed in this study, including “Ciri TBC Paru” showed consistently high correlation coefficients across all years (r = 0.382–0.731, all FDR-adjusted p < 0.05), while “Ciri Tuberkulosis” demonstrated increasingly strong correlations, reaching r = 0.731, FDR-adjusted p = 0.00005 in 2023. Likewise “Ciri TB Paru” remained significantly associated with TB case during every study year except 2022.

Keywords describing symptoms, transmission, prevention, and treatment also demonstrated robust associations with TB case. Searches for “Pencegahan TB” exhibited strong positive correlations across all five years, peaking at r = 0.704, FDR-adjusted p = 0.00008 in 2020. Similarly, “Gejala TB Paru”, “Penularan TB”, “Penularan TB Paru”, and treatment-related terms such as “Obat TBC Paru” consistently ranked among the most highly correlated search terms, with correlation coefficients frequently exceeding r = 0.65, FDR-adjusted p = 0.00023 in 2021.

Age-specific search terms also displayed substantial associations with TB case. Queries related to pediatric TB, including “TB Paru Anak”, “TBC Bayi”, and “TB Paru Bayi”, showed consistently moderate-to-strong positive correlations over multiple years. Notably, “TB Paru Anak” reached r = 0.707, FDR-adjusted p = 0.00008 in 2021, while “TB Paru Bayi” achieved r = 0.679, FDR-adjusted p = 0.00018 in 2023.

## Discussion

In general, this study showed a stable correlation between Google Trends and TB cases in Indonesia from 2019 to 2023, particularly for terms related to the characteristics, symptoms, transmission, and treatment. The terms tend to have moderate to strong and significantly positive correlations. This suggests that increasing cases in a region are accompanied by elevated public interest in searching for information on the signs, symptoms, and treatment. General terms such as “TB”, “TBC”, or “tuberkulosis” have weak and unstable correlations from yearly. This signified that technical terms are less sensitive in describing the dynamics of the caseload.

In 2020–2021, due to the COVID-19 pandemic, respiratory-related searches generally increased, but the public’s focus was on the virus, leading to a decrease in TB-related terms. However, terms such as “Flek paru-paru” and “Gejala TB paru” remained significantly correlated, reflecting strong sensitivity to the burden of this disease. Another study explained that the COVID-19 pandemic disrupted TB services globally, resulting in an 18% decrease in TB notifications in 2020 compared to 2019. Therefore, the system’s focus shifted to COVID-19 screening. ^29^ WHO also explained that the COVID-19 pandemic has affected access to TB diagnosis and service seeking due to public concern and the similar symptoms of the two diseases. ^30^ Research in Jiangsu Povince also suggests that the keywords TB symptoms and treatment are the strongest predictors of TB case trends. ^31^

Approaching the 2022–2023 period, the correlation pattern strengthened again and showed greater consistency compared to the pandemic period. A major recovery in TB detection and reporting after the COVID-19 disruption in 2022, with the number of diagnosed TB cases rising to 7.5 million, the highest since WHO began monitoring global TB. ^32^ Another study found that the most persistent keywords associated with TB case reports were TB symptoms and TB treatment. ^4^ In 2022, most terms related to the characteristics, symptoms, treatment, and transmission of TB continued to show significant positive correlations with moderate to strong strength across various provinces. This was evident in keywords such as “Ciri flek paru”, “Gejala TB Paru”, “Penularan TB Paru”, and “Obat flek paru”, which maintained statistical significance and relatively stable correlation values. Research from China suggests that search queries can serve as an additional surveillance system for TB and that search terms for TB symptoms and disease correlate significantly with actual TB incidence. ^33^ Terms related to children and infants, such as “TBC anak”, “TBC Paru anak”, and “Flek paru bayi”, also showed significant correlations. This suggests that TB-related searches in vulnerable age groups are increasingly sensitive to variations in caseload. This is in line with the estimated 1.3 million TB cases in children globally in 2022. Children are a major concern post-pandemic because many cases were previously undiagnosed. ^32^

In 2023, the strengthening trend in correlations became even more evident, particularly for clinical and specific terms. Several keywords, such “Ciri TB Paru”, “Ciri flek paru”, “Gejala flek paru”, “Penularan TB”, and “Obat TB paru”, showed significant correlations with consistent values in the moderate to strong category. Research using Google Trends in China concluded that disease-specific queries correlated more strongly than general terms, and that terms related to symptoms and diseases were better for TB surveillance than generic keywords. ^33^ This suggested that after a period of disruption to health services due to COVID-19, individuals are seeking more specific and targeted TB information. Conversely, general terms such as “TB” or “TBC” continue to show weak and inconsistent correlations, becoming less representative as a complementary exploratory surveillance tool. The 2022–2023 period confirms that symptomatic, diagnostic, and therapeutic keywords have higher sensitivity in reflecting variations in cases across regions than general terms. Previous research has shown that search terms related to TB symptoms, diagnosis and treatment have better predictive capabilities than general terms and more specific community search behavior. ^31^

A correlation analysis of Google Trends for TB cases in Indonesia shows that keywords based on symptoms, clinical characteristics, transmission, and treatment have a more stable relationship than general terms. As a result, the keywords are potentially being used as digital indicators to show spatio-temporal disease dynamics. Several infodemiology-based studies have shown that TB-related search volume on search engines significantly reduces cases and can reflect epidemiological trends. A study in South Africa showed a significant positive correlation between Google Trends search volume and reported incidence rates. Eight search terms showed moderate to strong associations, including “Tuberkulosis” and “TB,” as well as searches related to symptoms and diagnostic tests such as the *Mantoux test*. Furthermore, a strong correlation pattern was observed for terms related to comorbidities, particularly diabetes and HIV, reflecting the established pattern of HIV-TB infection in the region and increasing public awareness of the risk of diabetes to TB. ^34^

Another study used Google Trends to correlate measles clinical cases using Pearson correlation in 30 European countries and Japan. The results showed a very strong correlation at the regional level in developed countries, as observed in Okinawa Prefecture, Japan, during the 2017–2019 period. This is much higher than the correlation at the national level, proving that digital data searches are much more accurate in capturing signals of specific outbreaks in specific locations than on a broader scale. Search behavior is more sensitive to acute outbreaks that appear suddenly with many cases in a short period. Conversely, prolonged outbreaks with few cases per week often fail to be captured by Google Trends because individuals tend to no longer actively search for information when the disease is considered normal or not widespread. ^35^

Search for health information is often triggered by the development of disease symptoms that then trigger anxiety. When certain symptoms are experienced, search engines might be used to conduct initial identification before seeking professional medical help. ^36^ This behavior leads to a significant increase in search volume for symptom-related keywords, which often precedes official reports from health surveillance systems. The phenomenon suggests that digital search data can serve as an early indicator of public reaction to developing health threats. ^37^

Search volume tends to increase sharply in areas with a high disease burden or during outbreaks. In Indonesia, studies on dengue fever and COVID-19 have shown a strong correlation between the number of actual cases and search trends for keywords such as “ *symptoms*” or “ *transmission*”. ^38^ This linear pattern shows that individuals in affected areas are actively sourcing information for self-diagnosis or preventative measures. Therefore, a spike in searches in a specific region signals to health authorities the presence of potential disease hotspots requiring immediate intervention. ^20^

Pandemics, such as COVID-19, can create an overshadow effect that disrupts the seasonal patterns of other infectious diseases. During the peak of a pandemic, public attention and medical resources are highly focused on the novel virus. This leads to a decrease or change in search behavior toward other diseases, such as RSV or influenza. After pandemic mitigation measures are relaxed, search patterns and outbreaks of other diseases often reappear at irregular times and with greater intensity. This emphasizes the importance of using digital monitoring tools such as Google Trends to stay aware of multiple health risks amid the dominance of a single large outbreak. ^37^

The present study has several limitations. First, because this ecological study used aggregated province-level data, the observed correlations should not be interpreted as representing individual-level search behavior, indicating the potential for ecological fallacy. Second, Google Trends provides normalized RSVs rather than absolute search counts and relies on proprietary sampling and data processing methods, which may introduce variability in search volume estimates over time and should therefore be interpreted with caution. ^39^ Furthermore, online search behavior may be influenced by external factors, such as media coverage, public health campaigns, and major health events, rather than actual TB incidence. In addition, unequal internet penetration across Indonesian provinces may result in greater representation of urban populations than rural communities, while users’ search intentions and demographic characteristics cannot be determined from Google Trends data. Therefore, Google Trends should be considered a complementary surveillance tool rather than a replacement for conventional epidemiological surveillance systems. ^40^ Another important limitation is the potential confounding arising from symptom overlap between TB and other respiratory diseases. Symptoms such as persistent cough, fever, chest pain, and weight loss are not specific to TB and may also prompt searches related to influenza, COVID-19, pneumonia, or other respiratory infections. Although this study primarily included TB-specific search terms to reduce nonspecific searches, some symptom-related queries may still reflect public interest in other respiratory conditions. Consequently, Google Trends should be considered a complementary surveillance tool that captures public information-seeking behavior rather than a direct measure of TB incidence.

## Conclusion

Google Trends demonstrated a consistent and significant positive correlation with TB cases in Indonesia, from 2019 to 2023. Search terms based on commonly used clinical symptoms and treatment-related language were more informative than formal medical terminology, highlighting the importance of incorporating public search behavior into digital disease surveillance. Although the COVID-19 pandemic temporarily disrupted search patterns, these associations became stronger and more consistent after 2021. These findings support the potential integration of Google Trends into routine TB surveillance and public health decision-making, while future studies should further validate its application using additional epidemiological and digital data sources.

### Implications and recommendations

The results of this study provide an empirical basis for policymakers to integrate Google Trends data as an early warning system to complement conventional surveillance systems. Public health communication strategies should be optimized by using popular terms in the community, such as “Flek paru”. As a result, educational messages are more targeted and effective in encouraging early detection to achieve the national TB elimination target by 2030.

Future research should extend the present findings by employing multivariate models that incorporate province-level contextual factors, including internet accessibility, demographic and socioeconomic characteristics, healthcare service availability, and other determinants of TB burden. Such approaches may provide a more comprehensive understanding of the independent contribution of Google Trends search behavior to TB surveillance while accounting for potential confounding variables.

The search terms were generated using a systematic keyword construction strategy to comprehensively explore TB-related terminology commonly used by the Indonesian public when searching for TB-related information on Google. Four commonly used base terms referring to TB were first identified: “TB”, “TBC”, “Tuberkulosis”, and the colloquial term “Flek paru”. These base terms were then systematically combined with modifiers representing key domains of TB, including “paru”, “ciri”, “gejala”, “perobat”, “pencegahan”, “penularan”, “anak”, and “bayi”, thereby generating a comprehensive set of candidate search terms.

Only search terms that generated RSV data in Google Trends throughout the study period (2019–2023) were retained for analysis. The final dataset comprised 53 search terms, which were grouped into eight predefined categories: general TB terms, characteristics, symptoms, treatment, prevention, transmission, childhood TB, and infant TB ( Table 1).

Both medical terminology (e.g., “Tuberkulosis”) and commonly used lay expressions (e.g., “Flek paru”) were intentionally included because they are widely used by the Indonesian public to refer to pulmonary TB and therefore better represent real-world online health information-seeking behaviour. No formal expert validation or pilot testing was conducted because the objective of this study was to capture naturally occurring online search behaviour rather than to develop or validate a measurement instrument.

## Data Availability

Figshare: Public Search Behavior and Tuberculosis Cases in Indonesia 2019–2023: An Infodemiology Method Using Google Trends, https://doi.org/10.6084/m9.figshare.32905526 ^41^ The project contains the following underlying data:
• Data.xlsx. (Tuberculosis Case Data and Google Trends Search Data) Data are available under the terms of the Creative Commons Attribution 4.0 International license (CC-BY 4.0). Figshare: Public Search Behavior and Tuberculosis Cases in Indonesia 2019–2023: An Infodemiology Method Using Google Trends, https://doi.org/10.6084/m9.figshare.32905526. ^41^ This project sontaints the following extended data:
•
Table 3. Shapiro–Wilk Normality Test Results for Provincial Tuberculosis Cases and Google Trends Relative Search Volume (RSV), Indonesia, 2019–2023 Data are available under the terms of the Creative Commons Attribution 4.0 International license (CC-BY 4.0). Figshare: Public Search Behavior and Tuberculosis Cases in Indonesia 2019–2023: An Infodemiology Method Using Google Trends, https://doi.org/10.6084/m9.figshare.31969371. ^42^ This project sontaints the following extended data:
• Table 4. The relationship between the 53 TB search terms on Google Trends and number of TB cases in Indonesia 2019–2023 Data are available under the terms of the Creative Commons Attribution 4.0 International license (CC-BY 4.0).
