# Supplementary material for: Public Search Behavior and Tuberculosis Cases in Indonesia 2019-2023: An Infodemiology Study Using Google Trends 
Source: F1000Res. 2026 Sep 12;15:625. Originally published 2026 Apr 28. [Version 2] doi: 10.12688/f1000research.179772.2 (PMC13234547; doi:10.12688/f1000research.179772.2)
Supplement: Peer review history

# Reviewer report: Reviewer response for version 2

**DOI:** 10.5256/f1000research.206624.r520576

**Published:** 2026-09-23

**Authors:** Dr. Ravindra Nath

**Affiliations:** 1 Community Medicine, Autonomous State Medical College, Shahjahanpur, Moradabad, Uttar Pradesh, India

No further comments. The changes made by the authors are satisfactory.

Is the work clearly and accurately presented and does it cite the current literature?

Partly

If applicable, is the statistical analysis and its interpretation appropriate?

I cannot comment. A qualified statistician is required.

Are all the source data underlying the results available to ensure full reproducibility?

Yes

Is the study design appropriate and is the work technically sound?

Partly

Are the conclusions drawn adequately supported by the results?

Partly

Are sufficient details of methods and analysis provided to allow replication by others?

Partly

Reviewer Expertise:

Tuberculosis, Public Health, Infectious Diseases, Epidemiology, Health Communication, Diabetic Foot, Diabetes, Filariasis, Nutrition

I confirm that I have read this submission and believe that I have an appropriate level of expertise to confirm that it is of an acceptable scientific standard.

# Reviewer report: Reviewer response for version 2

**DOI:** 10.5256/f1000research.206624.r520578

**Published:** 2026-09-22

**Authors:** Miftahul Falah

**Affiliations:** 1 Universitas Muhammadiyah Tasikmalaya, West Java, Indonesia

After carefully reviewing the revised version, I confirm that the authors have adequately addressed and incorporated the comments and suggestions provided during the review process.

 The revisions are appropriate and have improved the clarity, quality, and overall presentation of the manuscript.

 Based on my assessment, the revised manuscript is now in accordance with the comments provided and is suitable for further consideration by the journal.

Is the work clearly and accurately presented and does it cite the current literature?

Partly

If applicable, is the statistical analysis and its interpretation appropriate?

Partly

Are all the source data underlying the results available to ensure full reproducibility?

Yes

Is the study design appropriate and is the work technically sound?

Yes

Are the conclusions drawn adequately supported by the results?

Yes

Are sufficient details of methods and analysis provided to allow replication by others?

Partly

Reviewer Expertise:

NA

I confirm that I have read this submission and believe that I have an appropriate level of expertise to confirm that it is of an acceptable scientific standard.

# Reviewer report: Reviewer response for version 1

**DOI:** 10.5256/f1000research.198320.r481277

**Published:** 2026-06-03

**Authors:** Falak Pahwa

**Affiliations:** 1 Microbiology, The University of Chicago, Illinois, Chicago, USA

Rahayu SR *et al. *have shown the use of Google Trends for public health surveillance for TB. This study demonstrates that Google Trends is a complementary indicator for TB trend monitoring and can provide timely and low-cost insights.

 However, I have a few comments that should be addressed prior to Indexing:

 Brief introductory note for Google Trends and its use for public health surveillance would strengthen the manuscript.

 TB transmission is often associated with shared and crowded environments, where marginalized populations are disproportionately represented. These groups may have limited access to smartphones or internet connectivity. Could the authors elaborate on whether Google Trends data might have underrepresented such populations?

 While rural settings are briefly discussed, a more detailed consideration of access disparities and their impact on data interpretation would strengthen the manuscript. TB shares several symptoms with other respiratory diseases. How do the authors account for potential confounding from searches related to other conditions with overlapping symptoms?

 Specifically, how is signal specificity ensured, and what strategies are used to minimize noise arising from non-TB-related searches?

 Please clarify whether the current analysis supports an early warning system, or whether it should be described more cautiously as exploratory infodemiological evidence.

 References 1 and 2 appear to be highly similar and could be consolidated to avoid redundancy. References 6-9 seem to direct to a single web page containing information for multiple years.

 These could potentially be combined into a single reference for clarity and conciseness.

 Although the authors state that age was not included as a factor in the analysis, exploring age and sex differences among TB patients could be a valuable avenue for future research.

Is the work clearly and accurately presented and does it cite the current literature?

Yes

If applicable, is the statistical analysis and its interpretation appropriate?

I cannot comment. A qualified statistician is required.

Are all the source data underlying the results available to ensure full reproducibility?

Partly

Is the study design appropriate and is the work technically sound?

Partly

Are the conclusions drawn adequately supported by the results?

Partly

Are sufficient details of methods and analysis provided to allow replication by others?

Partly

Reviewer Expertise:

Tuberculosis, Immunology, Aging

I confirm that I have read this submission and believe that I have an appropriate level of expertise to confirm that it is of an acceptable scientific standard.

# Response

**Published:** 2026-07-17

**Authors:** Sri Ratna Rahayu

**Affiliations:** Public Health, Universitas Negeri Semarang, Semarang, Central Java, Indonesia

1. Rahayu SR et al. have shown the use of Google Trends for public health surveillance for TB. This study demonstrates that Google Trends is a complementary indicator for TB trend monitoring and can provide timely and low-cost insights.

**Respond:**

 Thank you for the positive response to our research findings.

 2. However, I have a few comments that should be addressed prior to Indexing:Brief introductory note for Google Trends and its use for public health surveillance would strengthen the manuscript. TB transmission is often associated with shared and crowded environments, where marginalized populations are disproportionately represented. These groups may have limited access to smartphones or internet connectivity. Could the authors elaborate on whether Google Trends data might have underrepresented such populations?

**Respond:**

 Thank you for your suggestion.

 We have made improvements based on your suggestion in the Discussion section of the limitations paragraph.

 The present study has several limitations, including its reliance on internet access, which is not evenly distributed across Indonesia. This implies that Google Trends data may be more representative of the behavior of residents in urban areas than in rural areas. An important consideration when interpreting these findings is the potential underrepresentation of socially disadvantaged populations within Google Trends data.

 Tuberculosis disproportionately affects individuals living in overcrowded conditions, those with lower socioeconomic status, and populations with limited access to healthcare services. These vulnerable groups may also have reduced access to smartphones, internet connectivity, or digital health information, leading to lower representation in online search data.  Furthermore, the intent behind keyword searches cannot be fully ascertained, whether being performed by TB patients for treatment or by healthy individuals simply sourcing general information.

 Therefore, Google Trends should be considered a complementary surveillance approach alongside conventional epidemiological reporting systems rather than a substitute for them. The study also did not include user demographic factors such as age and education level, which may influence how search terms are formulated in search engines.

 3. While rural settings are briefly discussed, a more detailed consideration of access disparities and their impact on data interpretation would strengthen the manuscript. TB shares several symptoms with other respiratory diseases. How do the authors account for potential confounding from searches related to other conditions with overlapping symptoms?

**Respond:**

 Thank you for your suggestion. We have expanded the Limitation section to address both issues.

 unequal internet penetration across Indonesian provinces may result in greater representation of urban populations than rural communities, while users' search intentions and demographic characteristics cannot be determined from Google Trends data. Therefore, Google Trends should be considered a complementary surveillance tool rather than a replacement for conventional epidemiological surveillance systems

 The potential confounding arising from symptom overlap between TB and other respiratory diseases. Symptoms such as persistent cough, fever, chest pain, and weight loss are not specific to TB and may also prompt searches related to influenza, COVID-19, pneumonia, or other respiratory infections. Although this study primarily included TB-specific search terms to reduce nonspecific searches, some symptom-related queries may still reflect public interest in other respiratory conditions. Consequently, Google Trends should be considered a complementary surveillance tool that captures public information-seeking behavior rather than a direct measure of TB incidence.

 4. Specifically, how is signal specificity ensured, and what strategies are used to minimize noise arising from non-TB-related searches?Please clarify whether the current analysis supports an early warning system, or whether it should be described more cautiously as exploratory infodemiological evidence.

**Respond:**

 Thank you for this important comment. We agree that the specificity of signals derived from Google Trends cannot be fully ensured because the analysis was based entirely on publicly available aggregated data from the Indonesian Health Profile and Google Trends. Google Trends does not provide information regarding users' motivations or whether searches were performed by individuals affected by tuberculosis, healthcare professionals, students, or the general public. Consequently, non-TB-related search activity and external events may contribute to search volume and introduce background noise. To address this concern, we have revised the Limitations section to explicitly acknowledge the lack of signal specificity and recommend that future studies combine Google Trends data with additional surveillance sources and more advanced validation methods to improve signal specificity.

 In addition, we have revised the manuscript to avoid overstating the applicability of Google Trends as an early warning system. The findings are now presented more cautiously as exploratory infodemiological evidence demonstrating the potential utility of Google Trends for digital disease surveillance rather than as evidence supporting an operational early warning system. This clarification has also been incorporated into the Discussion and Limitations sections.

 5. References 1 and 2 appear to be highly similar and could be consolidated to avoid redundancy. References 6-9 seem to direct to a single web page containing information for multiple years. These could potentially be combined into a single reference for clarity and conciseness.

**Respond:**

 Thank you for your suggestion. We've made improvements based on your suggestions.

 6. Although the authors state that age was not included as a factor in the analysis, exploring age and sex differences among TB patients could be a valuable avenue for future research.

**Respond:**

 Thank you for this valuable suggestion. We agree that exploring age- and sex-specific differences in tuberculosis-related information-seeking behavior would provide additional insights. However, the present study was designed as an ecological analysis using aggregated provincial-level tuberculosis case data and Google Trends Relative Search Volume (RSV). Consequently, age- and sex-stratified analyses were beyond the scope of the current study because individual-level demographic information is not available in Google Trends data.

 To address the reviewer's suggestion, we have added this point to the Limitations and Future Research section, highlighting that future studies should investigate age- and sex-specific search behaviors using more detailed epidemiological datasets where such information is available.

# Reviewer report: Reviewer response for version 1

**DOI:** 10.5256/f1000research.198320.r485774

**Published:** 2026-06-02

**Authors:** Miftahul Falah

**Affiliations:** 1 Universitas Muhammadiyah Tasikmalaya, West Java, Indonesia

Review Report : 

**1. Title**

 The title is informative; however, the phrase *“An Infodemiology Method Using Google Trends”* is grammatically awkward. Consider revising it to:

*“An Infodemiology Study Using Google Trends”*

 or

*“An Infodemiology Approach Using Google Trends”*

**2. Abstract (Page 1–2)**

**Background Section (Page 1, Paragraph 1)**

 The sentence:

 “Tuberculosis (TB) is a major health challenge in Indonesia, which ranks second globally in 2024.”

 should be revised for clarity because it implies Indonesia itself is “the challenge.” Suggested revision:

 “Tuberculosis (TB) remains a major public health challenge in Indonesia, which ranked second globally in TB burden in 2024.”

**Methods Section (Page 1, Paragraph 2)**

 The statement:

 “Case data were normalized (0–100) to reflect the Relative Search Volume (RSV).”

 requires additional explanation. The abstract should briefly clarify *why* normalization was necessary and how it was performed?

**Results Section (Page 2)**

 The results are excessively detailed for an abstract. Listing many correlation coefficients makes the abstract difficult to read. Please summarize only the major findings and report the strongest correlations more concisely.

 For example:

 “Several symptom- and treatment-related search terms showed consistently strong positive correlations with TB case reports across provinces.”

**3. Introduction (Page 3)**

**Paragraph 1 (Page 3)**

 The sentence:

 “However, control remains at 12%, a value far from the target of 50% by 2025.”

 is unclear because “control” is not specifically defined. Please clarify whether this refers to case detection, treatment success, or another TB control indicator.

**Paragraph 1 (Page 3)**

 The statement:

 “This gap shows that sufferers and the public tend to express their health awareness regarding TB through searching for information in digital spaces…”

 is speculative and unsupported by evidence. The authors should provide a supporting reference or rephrase more cautiously.

 Suggested revision:

 “This gap may indicate increased public reliance on digital platforms to seek TB-related health information.”

**Final Paragraph of Introduction (Page 3)**

 The research objectives are not explicitly stated. Please clearly state:

1. The primary objective.
2. Secondary objectives.
3. The novelty of study unclear

A dedicated final paragraph for study objectives is strongly recommended.

**4. Methods Section (Pages 3–4)**

**Study Design (Page 3, “Study Type and Design”)**

 The manuscript describes the study as:

 “cross-sectional”

 However, the analysis uses multi-year provincial data (2019–2023), which is more consistent with an ecological longitudinal or repeated cross-sectional design.

 Please revise and justify the study design terminology appropriately.

**Data Source Section (Page 4)**

 The authors should clarify:

- Whether Google Trends data were extracted using:
  - “search term” or “topic”
  - “health category” or “all categories”
  - “web search” or other search types
- Whether the searches were conducted in Indonesian language only.

These details are essential for reproducibility.

**Study Variables (Page 4)**

 The explanation of the 53 keywords is insufficient.

 Please explain:

- How the keywords were generated.
- Whether experts validated them.
- Whether pilot testing was conducted.
- Why certain colloquial terms such as “flek paru” were included.

**Data Normalization (Page 4)**

 The normalization formula is incomplete and poorly formatted:

 “Normalization Formula ¼ Highest scale in this case 100…”

 This section requires major revision because the mathematical explanation is unclear.

 Please:

- Rewrite the formula correctly.
- Explain each variable.
- Explain why normalization was necessary before Spearman analysis.

**5. Statistical Analysis (Page 4)**

**Statistical Testing**

 The manuscript only reports Spearman correlation coefficients and p-values.

 Please additionally report:

- Confidence intervals.
- Interpretation criteria with supporting references.
- Whether assumptions for Spearman correlation were checked.

**Multiple Comparisons Issue**

 Since 53 terms × 5 years were analyzed, there is a high risk of Type I error.

 The authors should discuss:

- Bonferroni correction,
- False Discovery Rate (FDR), or
- Justification for not adjusting multiple comparisons.

This is a major methodological concern.

**6. Results Section (Pages 4–6)**

**Presentation of Results**

 The Results section is overly narrative and repetitive.

 For example, the repeated sentence structure:

 “a strong and positive correlation was observed…”

 appears excessively throughout Pages 4–6.

 Please summarize findings more efficiently using:

- tables,
- heatmaps,
- trend figures,
- or clustered correlation plots.

**Table 1 (Page 5)**

 There are inconsistencies in terminology:

- “Lung spot”
- “Lungs spot”

Please standardize terminology throughout the manuscript.

**Table 2 (Page 5)**

 Decimal notation uses commas instead of periods:

 “0,70–0,89”

 Please standardize according to journal style:

 “0.70–0.89”

**7. Discussion Section (Pages 6–7)**

**Paragraph 1 (Page 6)**

 The sentence:

 “technical terms are less sensitive in describing the dynamics of the caseload”

 needs stronger scientific explanation. Why were non-medical terms more strongly correlated? Consider discussing:

- health literacy,
- public language preferences,
- cultural terminology,
- digital search behavior theory.

**COVID-19 Discussion (Page 6)**

 The discussion regarding COVID-19 overshadowing TB searches is interesting but lacks supporting Indonesian references. Please strengthen this section with local or regional evidence.

**Discussion Redundancy**

 Several paragraphs merely restate the Results section without deeper interpretation.

 Please reduce repetition and focus on:

- implications,
- mechanisms,
- comparison with previous studies,
- theoretical explanations.

**8. Limitations (Page 7)**

 The limitation section is currently too brief.

 Please additionally discuss:

- Ecological fallacy risk.
- Google Trends algorithm instability.
- Influence of media coverage.
- Unequal internet penetration across provinces.
- Potential bias from urban populations dominating online searches.

This section needs stronger critical reflection.

**9. Conclusion (Page 7)**

 The conclusion is too long and partially repetitive.

 Please shorten and focus on:

- the most important findings,
- practical implications,
- future surveillance potential.

Avoid re-discussing detailed results.

Is the work clearly and accurately presented and does it cite the current literature?

Partly

If applicable, is the statistical analysis and its interpretation appropriate?

Partly

Are all the source data underlying the results available to ensure full reproducibility?

Yes

Is the study design appropriate and is the work technically sound?

Yes

Are the conclusions drawn adequately supported by the results?

Yes

Are sufficient details of methods and analysis provided to allow replication by others?

Partly

Reviewer Expertise:

Tuberculosis, Family Support, medication compliance, TB promotion

I confirm that I have read this submission and believe that I have an appropriate level of expertise to confirm that it is of an acceptable scientific standard, however I have significant reservations, as outlined above.

# Response

**Published:** 2026-07-17

**Authors:** Sri Ratna Rahayu

**Affiliations:** Public Health, Universitas Negeri Semarang, Semarang, Central Java, Indonesia

1. Title. The title is generally informative; however, the phrase “An Infodemiology Method Using Google Trends” is grammatically awkward. It is recommended to revise it to: “An Infodemiology Study Using Google Trends” or “An Infodemiology Approach Using Google Trends”

**Respond:**

 Thank you for your valuable suggestion. We have revised the title to improve its clarity and grammatical accuracy by replacing "An Infodemiology Method Using Google Trends" with "An Infodemiology Study Using Google Trends."

 2. Abstract (Page 1–2). Background Section (Page 1, Paragraph 1). The sentence: “Tuberculosis (TB) is a major health challenge in Indonesia, which ranks second globally in 2024.” should be revised for clarity because it implies Indonesia itself is “the challenge.” Suggested revision: “Tuberculosis (TB) remains a major public health challenge in Indonesia, which ranked second globally in TB burden in 2024.”

 Methods Section (Page 1, Paragraph 2). The statement: “Case data were normalized (0–100) to reflect the Relative Search Volume (RSV).” requires additional explanation. The abstract should briefly clarify why normalization was necessary and how it was performed?Results Section (Page 2). The results are excessively detailed for an abstract. Listing many correlation coefficients makes the abstract difficult to read. Please summarize only the major findings and report the strongest correlations more concisely. For example: “Several symptom- and treatment-related search terms showed consistently strong positive correlations with TB case reports across provinces.”

**Respond:**

 Thank you for your suggestion and important observation.

 We've made improvements based on your suggestions. We agree that the original terminology did not adequately reflect the structure of our dataset. Accordingly, we have revised the manuscript to describe the study as an ecological repeated cross-sectional study, as the unit of analysis was the province and the analyses were conducted separately using annual provincial data from 2019 to 2023 rather than following the same individuals over time. We have also revised the description in the Methods section to better justify the study design.

 3. Introduction (Page 3). Paragraph 1 (Page 3). The sentence: “However, control remains at 12%, a value far from the target of 50% by 2025.” is unclear because “control” is not specifically defined. Please clarify whether this refers to case detection, treatment success, or another TB control indicator.Paragraph 1 (Page 3). The statement: “This gap shows that sufferers and the public tend to express their health awareness regarding TB through searching for information in digital spaces…” is speculative and unsupported by evidence. The authors should provide a supporting reference or rephrase more cautiously. Suggested revision: “This gap may indicate increased public reliance on digital platforms to seek TB-related health information.”

 Final Paragraph of Introduction (Page 3). The research objectives are not explicitly stated. Please clearly state: The primary objective. Secondary objectives. The novelty of study unclear. A dedicated final paragraph for study objectives is strongly recommended.

**Respond:**

 Thank you for your suggestion.

 We've made improvements based on your suggestions.

 In the last paragraph of the introduction, we have explained the main objective, secondary objectives, and novelty of this research briefly but clearly.

 “This study was to evaluate the correlation between Google Trends search volumes and reported TB cases in Indonesia from 2019 to 2023 using various Indonesian-language search terms, encompassing disease names, symptoms, clinical characteristics, treatment, prevention, transmission, and pediatric TB. Correlation of multi-term data with the number of TB cases reported in the official annual Indonesian Health Profile over the past five years, hence provided a basis for evaluating the potential frequency of digital searches as a complementary indicator for more responsive epidemiological surveillance.

 Unlike previous studies that typically relied on a limited number of generic keywords, this study identifies culturally relevant search terms that more accurately reflect TB trends in Indonesia and provides evidence to support the development of locally adapted digital disease surveillance strategies.

 Through an infodemiology method, the results of this study are expected to identify the most trending information in real-life cases, thereby providing an empirical basis for the development of an early warning system and targeted health communication in Indonesia.”

 4. Methods Section (Pages 3–4). Study Design (Page 3, “Study Type and Design”). The manuscript describes the study as: “cross-sectional” However, the analysis uses multi-year provincial data (2019–2023), which is more consistent with an ecological longitudinal or repeated cross-sectional design. Please revise and justify the study design terminology appropriately.Data Source Section (Page 4). The authors should clarify: Whether Google Trends data were extracted using: “search term” or “topic” ,“health category” or, “all categories”, “web search” or other search types. Whether the searches were conducted in Indonesian language only. These details are essential for reproducibility.

 Study Variables (Page 4). The explanation of the 53 keywords is insufficients. Please explain: How the keywords were generated. Whether experts validated them. Whether pilot testing was conducted. Why certain colloquial terms such as “flek paru” were included.

 Data Normalization (Page 4). The normalization formula is incomplete and poorly formatted: “Normalization Formula ¼ Highest scale in this case 100…”. This section requires major revision because the mathematical explanation is unclear. Please: Rewrite the formula correctly. Explain each variable. Explain why normalization was necessary before Spearman analysis.

**Respond:**

 - Thank you for this important observation. We agree that the original terminology did not adequately reflect the structure of our dataset. Accordingly, we have revised the manuscript to describe the study as an ecological repeated cross-sectional study, as the unit of analysis was the province and the analyses were conducted separately using annual provincial data from 2019 to 2023 rather than following the same individuals over time. We have also revised the description in the Methods section to better justify the study design.

 - We have revised the Data Source section to explicitly describe the Google Trends extraction strategy. The revised manuscript now specifies that Google Trends data were extracted using search terms (rather than topics), with the geographic filter set to Indonesia, the search category set to All Categories, the search type set to Web Search, and all queries entered in the Indonesian language. We also clarified that data were extracted separately for each study year (2019–2023), with each search term generating an annual CSV file containing Interest by subregion RSV values for all 34 Indonesian provinces.

 - We have substantially expanded the Keyword Selection section to improve transparency and reproducibility. Specifically, four base terms referring to tuberculosis (TB, TBC, tuberkulosis, and the colloquial term flek paru) were systematically combined with modifiers representing key TB domains, including pulmonary disease, characteristics, symptoms, treatment, prevention, transmission, children, and infants. We also clarified that only search terms generating Relative Search Volume (RSV) data in Google Trends during the study period (2019–2023) were retained, resulting in a final set of 53 search terms grouped into eight categories. Furthermore, we explained that colloquial terms such as flek paru were intentionally included because they are widely used by the Indonesian public to refer to pulmonary tuberculosis and therefore better reflect real-world online search behaviour. Finally, we clarified that no formal expert validation or pilot testing was performed because the objective of this study was to capture naturally occurring online search behaviour rather than to develop or validate a measurement instrument.

 - We have substantially revised the Data Normalization and Conversion section to improve its clarity and reproducibility. Specifically, we reformatted the normalization formula using standard mathematical notation and explicitly defined each variable used in the equation. We also added a detailed explanation describing the rationale for normalization, clarifying that the official provincial TB case counts were reported as absolute numbers, whereas Google Trends Relative Search Volume (RSV) is expressed on a standardized 0–100 scale. Therefore, annual maximum normalization was applied to rescale the provincial TB case counts to the same 0–100 interval as the RSV data, facilitating direct comparison and interpretation while preserving the relative differences among provinces within each study year. In addition, we included a worked example using the 2019 provincial TB case data (West Java and Aceh) to illustrate the normalization procedure step by step and clarified that this process was repeated independently for each study year (2019–2023) before pairing the normalized TB case scores with the corresponding Google Trends RSV values for Spearman correlation analysis.

 5. Statistical Analysis (Page 4). Statistical Testing. The manuscript only reports Spearman correlation coefficients and p-values. Please additionally report: Confidence intervals. Interpretation criteria with supporting references. Whether assumptions for Spearman correlation were checked. Multiple Comparisons Issue. Since 53 terms × 5 years were analyzed, there is a high risk of Type I error.The authors should discuss: Bonferroni correction, False Discovery Rate (FDR), or Justification for not adjusting multiple comparisons. This is a major methodological concern.

**Respond:**

 Thank you very much for this valuable methodological comment. We have carefully revised the Statistical Analysis section to address all of the reviewer's concerns.

 First, regarding confidence intervals, IBM SPSS Statistics Version 25 does not provide confidence intervals for Spearman's rank correlation coefficients in its standard correlation procedure. Therefore, only Spearman's correlation coefficients (ρ) and corresponding P-values are reported. This limitation has been acknowledged, and the statistical outputs generated by SPSS were used consistently throughout the analysis.

 Second, we have now added the interpretation criteria for Spearman's correlation coefficients in the Methods section. The interpretation follows the recommendations of Schober and Schwarte (2018), which provide guidance on the appropriate interpretation of correlation coefficients in biomedical research. This reference has also been included in the reference list.

 Schober P, Schwarte LA. Correlation coefficients: Appropriate use and interpretation. Anesth Analg. 2018;126(5):1763–1768. https://doi.org/10.1213/ANE.0000000000002864

 Third, the assumptions for Spearman's correlation were verified prior to the correlation analysis. Normality was assessed using the Shapiro–Wilk test because each annual dataset consisted of 34 provincial observations. The results indicated that the variables were not normally distributed across all study years; therefore, Spearman's rank correlation was considered the appropriate non-parametric statistical test. The normality test results have been added as Supplementary Table 3  and are now available in the supplementary materials deposited in Figshare.

 The Methods section has been revised accordingly.

 Fourth, we agree that conducting 53 correlation tests annually increases the risk of Type I error due to multiple comparisons in Table 4. To address this issue, we have revised the statistical analysis by applying the Benjamini–Hochberg False Discovery Rate (BH-FDR) correction separately for each study year. For each year, the 53 raw P-values obtained from the Spearman correlation analyses were ranked in ascending order, and FDR-adjusted P-values were calculated according to the Benjamini–Hochberg procedure. The adjusted P-values were subsequently reported in both the revised Results section and the updated supplementary table. The complete BH-FDR calculation procedure has also been described in the Statistical Analysis subsection of the Methods.

 6. Results Section (Pages 4–6). Presentation of Results. The Results section is overly narrative and repetitive. For example, the repeated sentence structure: “a strong and positive correlation was observed…” appears excessively throughout Pages 4–6. Please summarize findings more efficiently using: tables, heatmaps, trend figures, or clustered correlation plots. Table 1 (Page 5). There are inconsistencies in terminology: “Lung spot”

 “Lungs spot” Please standardize terminology throughout the manuscript.

 Table 2 (Page 5). Decimal notation uses commas instead of periods: “0,70–0,89” Please standardize according to journal style: “0.70–0.89”. Accordingly, the manuscript now reports both the raw P-values and the FDR-adjusted P-values, thereby reducing the likelihood of false-positive findings while maintaining adequate statistical power for multiple hypothesis testing.

**Respond:**

 Thank you for your constructive comments regarding the presentation of the Results section and the formatting of the tables. Following your suggestion, we have revised the Results section by reducing repetitive narrative descriptions and emphasizing the principal findings. Furthermore, Table 4 has been expanded to present the Spearman correlation coefficients (Spearman's r), raw p-values, and False Discovery Rate (FDR)-adjusted p-values for all 53 tuberculosis-related search terms across the five study years (2019–2023). Although graphical visualizations such as heatmaps or clustered correlation plots may be useful for summarizing large datasets, we believe that, in the present study, a comprehensive table provides greater clarity because each of the 53 search terms requires simultaneous presentation of three statistical measures (Spearman's r, raw p-value, and FDR-adjusted p-value) for five separate years. Presenting these data in graphical form would substantially reduce readability and prevent readers from identifying the exact statistical estimates for each search term. Therefore, we retained the tabular presentation while simplifying the accompanying narrative to improve readability.

 Regarding the terminology "Lung spot" and "Lungs spot," these are not inconsistencies but intentional translations of two different Indonesian search queries included in Google Trends. Specifically, "Flek paru" and "Flek paru-paru" represent distinct search terms that were entered separately into Google Trends and therefore generated independent Relative Search Volume (RSV) results. Because the objective of this study was to evaluate actual public search behavior, both terms were intentionally analyzed and reported separately throughout the manuscript.

 Concerning the decimal notation, the current manuscript was prepared before selecting the target journal and therefore follows our internal formatting convention. The decimal separator will be revised to fully comply with the formatting guidelines of the target journal upon submission.

 7. Discussion Section (Pages 6–7). Paragraph 1 (Page 6). The sentence: “technical terms are less sensitive in describing the dynamics of the caseload” needs stronger scientific explanation. Why were non-medical terms more strongly correlated? Consider discussing: health literacy, public language preferences, cultural terminology, digital search behavior theory.COVID-19 Discussion (Page 6). The discussion regarding COVID-19 overshadowing TB searches is interesting but lacks supporting Indonesian references. Please strengthen this section with local or regional evidence.

 Discussion Redundancy. Several paragraphs merely restate the Results section without deeper interpretation. Please reduce repetition and focus on:

 implications, mechanisms, comparison with previous studies, theoretical explanations.

**Respond:**

 Thank you for your suggestion.

 We've made improvements based on your suggestions.

 8. Limitations (Page 7). The limitation section is currently too brief. Please additionally discuss: Ecological fallacy risk. Google Trends algorithm instability. Influence of media coverage. Unequal internet penetration across provinces. Potential bias from urban populations dominating online searches. This section needs stronger critical reflection.

**Respond:**

 Thank you for this valuable suggestion. We agree that the original Limitations section required a more comprehensive discussion. Accordingly, we have expanded this section by discussing the potential for ecological fallacy, the methodological limitations of Google Trends related to its proprietary sampling and data processing methods, the influence of media coverage on search behavior, unequal internet penetration across Indonesian provinces, and the potential overrepresentation of urban populations in online searches. We have also strengthened the critical reflection by clarifying that Google Trends should be interpreted as a complementary tool for tuberculosis surveillance rather than a substitute for conventional epidemiological surveillance.

 9. Conclusion (Page 7). The conclusion is too long and partially repetitive. Please shorten and focus on: the most important findings, practical implications, future surveillance potential. Avoid re-discussing detailed results. Is the work clearly and accurately presented and does it cite the current literature?

**Respond:**

 Thank you for your valuable suggestion. We have revised the Conclusion section by substantially shortening it and removing repetitive descriptions of the study results. The revised conclusion now emphasizes the principal findings, highlights the practical implications of using Google Trends as a complementary tool for tuberculosis surveillance in Indonesia, and outlines its potential for future public health surveillance. We have also avoided repeating detailed results that are already presented in the Results and Discussion sections.

# Reviewer report: Reviewer response for version 1

**DOI:** 10.5256/f1000research.198320.r481276

**Published:** 2026-06-02

**Authors:** Saidah Rauf

**Affiliations:** 1 Poltekkes Kemenkes Maluku, Ambon, Indonesia

The study design is appropriate, and the work is technically sound. The manuscript also demonstrates novelty, particularly in its attempt to link Google Trends terminology with TB burden at the provincial level. In addition, the methods and analytical procedures are described in sufficient detail to allow replication by other researchers. The underlying source data appear to be adequately available to support reproducibility, and the conclusions are generally supported by the results. Nevertheless, minor refinements in the discussion would further strengthen the manuscript. Several points should be highlighted. First, the discussion requires stronger support from current and relevant literature, particularly in paragraphs 2–5, to ensure that the interpretation is evidence-based and does not appear speculative. Second, although the statistical analysis is generally appropriate, it could be further strengthened by extending the analysis to a multivariate approach, considering the use of Google Trends terminology and its significant association with TB burden across more than two provinces.

Is the work clearly and accurately presented and does it cite the current literature?

Partly

If applicable, is the statistical analysis and its interpretation appropriate?

Partly

Are all the source data underlying the results available to ensure full reproducibility?

Yes

Is the study design appropriate and is the work technically sound?

Yes

Are the conclusions drawn adequately supported by the results?

Yes

Are sufficient details of methods and analysis provided to allow replication by others?

Yes

Reviewer Expertise:

Tuberculosis, Neuroscience, Neurondocrine, Physiology

I confirm that I have read this submission and believe that I have an appropriate level of expertise to confirm that it is of an acceptable scientific standard, however I have significant reservations, as outlined above.

# Response

**Published:** 2026-06-29

**Authors:** Sri Ratna Rahayu

**Affiliations:** Public Health, Universitas Negeri Semarang, Semarang, Central Java, Indonesia

Thank you for this thoughtful suggestion. We agree that multivariate analyses may provide additional insights by accounting for potential confounding factors. However, the primary objective of this study was to evaluate the bivariate association between Google Trends Relative Search Volume (RSV) and provincial tuberculosis (TB) case counts using Spearman correlation. Because the study was designed as an exploratory correlation analysis and no additional province-level covariates (e.g., internet penetration, socioeconomic indicators, or healthcare access) were included in the study design, multivariate modelling was beyond the scope of the present study. Nevertheless, we have acknowledged this point in the Discussion as a recommendation for future research, suggesting that multivariate analyses incorporating relevant contextual variables could further strengthen the understanding of the relationship between online search behavior and TB burden.

# Reviewer report: Reviewer response for version 1

**DOI:** 10.5256/f1000research.198320.r485776

**Published:** 2026-05-27

**Authors:** Dr. Ravindra Nath

**Affiliations:** 1 Community Medicine, Autonomous State Medical College, Shahjahanpur, Moradabad, Uttar Pradesh, India

**Is the work clearly and accurately presented and does it cite the current literature?**

**(Partly)**

1. Please strengthen the Introduction by explaining more clearly why Google Trends is expected to reflect tuberculosis burden in the Indonesian setting.
2. The Discussion section contains some repetitive interpretation of symptom-related and “lung spot” search terms. Consider tightening these paragraphs for better readability.
3. Please include a few more international studies on TB-related infodemiology/digital surveillance to provide broader global context beyond Indonesia and South Africa.
4. Some conclusions in the manuscript appear stronger than the presented findings. Consider using slightly more cautious language throughout the Discussion and Conclusion sections.

**Are sufficient details of methods and analysis provided to allow replication by others?**

**(Partly)**

1. Please specify the exact Google Trends extraction settings used, including:
  - geographic filters,
  - language settings,
  - search category,
  - search type.
2. Clarify whether RSV values were extracted separately for each province and keyword before analysis.
3. The normalization formula should be rewritten more clearly to improve reproducibility.
4. Consider adding the complete extraction strategy as supplementary material.

**Are all the source data underlying the results available to ensure full reproducibility?**

**(Yes)**

1. The availability of both underlying and extended datasets through Figshare is a major strength of the manuscript and supports transparency and reproducibility.

**Are the conclusions drawn adequately supported by the results?**

**(Partly)**

1. The conclusions are broadly supported by the observed correlations.
2. However, statements suggesting Google Trends as an “early warning system” should be phrased more cautiously, as the current analysis demonstrates association rather than predictive validation.
3. Consider framing Google Trends as a complementary exploratory surveillance tool rather than a definitive epidemiological monitoring system.

Is the work clearly and accurately presented and does it cite the current literature?

Partly

If applicable, is the statistical analysis and its interpretation appropriate?

I cannot comment. A qualified statistician is required.

Are all the source data underlying the results available to ensure full reproducibility?

Yes

Is the study design appropriate and is the work technically sound?

Partly

Are the conclusions drawn adequately supported by the results?

Partly

Are sufficient details of methods and analysis provided to allow replication by others?

Partly

Reviewer Expertise:

Tuberculosis, Public Health, Infectious Diseases, Epidemiology

I confirm that I have read this submission and believe that I have an appropriate level of expertise to confirm that it is of an acceptable scientific standard, however I have significant reservations, as outlined above.

# Response

**Published:** 2026-07-17

**Authors:** Sri Ratna Rahayu

**Affiliations:** Public Health, Universitas Negeri Semarang, Semarang, Central Java, Indonesia

1. Please strengthen the Introduction by explaining more clearly why Google Trends is expected to reflect tuberculosis burden in the Indonesian setting.

 Respond:

 Thank you for your suggestion. We have revised the Introduction by providing a clearer rationale for why Google Trends is expected to reflect tuberculosis burden in the Indonesian setting and strengthened the supporting explanation accordingly.

 2. The Discussion section contains some repetitive interpretation of symptom-related and “lung spot” search terms. Consider tightening these paragraphs for better readability.

 Respond:

 Thank you for your suggestion.

 We've made improvements based on your suggestions.

 3. Please include a few more international studies on TB-related infodemiology/digital surveillance to provide broader global context beyond Indonesia and South Africa.

 Respond:

 Thank you for your suggestion. We have revised the Introduction by incorporating additional international evidence on TB-related digital surveillance to provide a broader global context. Specifically, we added recent studies describing the application of digital health technologies for tuberculosis surveillance and the use of Google Trends as a complementary tool for monitoring tuberculosis trends.

 4. Some conclusions in the manuscript appear stronger than the presented findings. Consider using slightly more cautious language throughout the Discussion and Conclusion sections.

 Respond:

 Thank you for your suggestion.

 We've made improvements based on your suggestions.

 5. Please specify the exact Google Trends extraction settings used, including: geographic filters, language settings, search category, search type.

 Respond:

 Thank you for this valuable suggestion. We agree that providing detailed Google Trends extraction settings improves the reproducibility of the study. We have revised the Methods section to explicitly describe the Google Trends extraction parameters. Specifically, the geographic filter was set to Indonesia, the search terms were entered in the Indonesian language, the search category was set to All Categories, and the search type was Web Search. These details have now been added to the revised manuscript.

 6. Clarify whether RSV values were extracted separately for each province and keyword before analysis.

 Respond:

 Thank you for this helpful comment. We have clarified this procedure in the revised Methods section. Relative Search Volume (RSV) values were extracted separately for each of the 53 TB-related search terms and for each study year (2019–2023). For every search term, Google Trends generated a separate annual CSV file containing Interest by subregion results, which provided RSV values for all 34 Indonesian provinces. These province-specific RSV values were then matched with the corresponding annual provincial TB case data from the Indonesian Health Profile before conducting the Spearman correlation analysis.

 7. The normalization formula should be rewritten more clearly to improve reproducibility.

 Respond:

 Thank you for this valuable comment. We agree that additional methodological detail improves the reproducibility of the study. Therefore, we have substantially revised the Data Normalization and Conversion subsection to provide a clearer description of the normalization procedure. Specifically, we clarified that provincial tuberculosis (TB) case counts were normalized separately for each study year (2019–2023) using annual maximum normalization to align the data with the 0–100 Relative Search Volume (RSV) scale of Google Trends. We also explicitly described the normalization formula, defined the annual reference value used for scaling, and included a worked example demonstrating the calculation process. These additions provide sufficient detail to enable other researchers to reproduce the normalization procedure and subsequent statistical analysis.

 8. Consider adding the complete extraction strategy as supplementary material.

 Respond:

 Thank you for this valuable suggestion. We agree that a transparent description of the data extraction process is important for reproducibility. Because the extraction procedure was relatively straightforward and relied entirely on publicly available Google Trends and Indonesian Health Profile data, we expanded the description directly in the Methods section rather than preparing supplementary material.

 Specifically, we clarified the Google Trends extraction settings (Web Search, Indonesia, All Categories, annual time interval, and province-level Interest by subregion data). We also explained that, for each study year (2019–2023), all 53 tuberculosis-related search terms were queried individually, with one CSV file generated for each search term. Each CSV contained Relative Search Volume (RSV) data for all 34 Indonesian provinces, which were subsequently matched with the corresponding provincial TB case data from the Indonesian Health Profile for the same year prior to Spearman correlation analysis.

 To further improve reproducibility, we also expanded the description of the normalization procedure by providing the normalization formula and a worked example using Aceh Province in 2019, illustrating how provincial TB case counts were rescaled to the 0–100 scale before being paired with Google Trends RSV data. We believe these additions provide sufficient methodological detail to allow readers to reproduce the analytical workflow without requiring supplementary material.

 9. However, statements suggesting Google Trends as an “early warning system” should be phrased more cautiously, as the current analysis demonstrates association rather than predictive validation.

 Respond:

 Thank you for your suggestion.

 We've made improvements based on your suggestions.

 10. Consider framing Google Trends as a complementary exploratory surveillance tool rather than a definitive epidemiological monitoring system.

 Respond:

 Thank you for your suggestion.

 We've made improvements based on your suggestions.
